# *Rhododendron jiucaipingensis* (Ericaceae), a new species from Guizhou, China

**DOI:** 10.3897/phytokeys.275.183519

**Published:** 2026-05-19

**Authors:** Hong-Fen Hu, Jian Xu, Ming-Tai An, Jiang-Hong Yu, Xing Lu

**Affiliations:** 1 School of Ecology and Nature Conservation, Beijing Forestry University, CN-100083 Beijing, China Guizhou Botanical Garden Guiyang China; 2 Guizhou Botanical Garden, CN-550000 Guiyang, China College of Forestry, Guizhou University Guiyang China; 3 College of Forestry, Guizhou University, CN-550025 Guiyang, China Key Laboratory of Plant Resource Conservation and Germplasm Innovation in Moun-tainous Region (Ministry of Education), College of Life Sciences/Institute of Agro-bioengineering, Guizhou University Guiyang China; 4 Key Laboratory of Plant Resource Conservation and Germplasm Innovation in Moun-tainous Region (Ministry of Education), College of Life Sciences/Institute of Agro-bioengineering, Guizhou University, CN-550025 Guiyang, China School of Ecology and Nature Conservation, Beijing Forestry University Beijing China; 5 People's Government of Xingfa Miao, Yi and Hui Ethnic Township, CN-553200, Hezhang, China People's Government of Xingfa Miao, Yi and Hui Ethnic Township Hezhang China

**Keywords:** Ericaceae, Flora of China, morphology, phylogeny, taxonomy

## Abstract

*Rhododendron jiucaipingensis* Jian Xu & M. T. An, **sp. nov**. (Ericaceae), a new species of *R.* subgen. *Hymenanthes*, subsect. *Argyrophylla*, from northwestern Guizhou, China, is described and illustrated. It is an evergreen shrub 1.5–3.0 m tall, characterized by green, densely white-tomentose current-year branchlets, leaves crowded at branch apices (5.5–9.5 × 2.5–4.0 cm) with 8–11 pairs of lateral veins, 10 stamens, and cylindric capsules 1.2–2.0 cm long. The new species is morphologically most similar to *R. hypoglaucum* and *R. argyrophyllum*, but it is readily distinguished by having densely white-tomentose current-year branchlets, fewer lateral veins (8–11 pairs), a double-layered white indumentum on the abaxial surfaces of young leaves, and 10 stamens. Phylogenetic analysis based on a whole-genome SNP dataset robustly resolves *R. jiucaipingensis* as a distinct lineage within subsect. *Argyrophylla*, positioned as sister to the monophyletic clade of five congeneric species with high support (SH-aLRT = 100%, UFBoot = 100%). This molecular evidence, congruent with diagnostic morphological characters, supports the recognition of *R. jiucaipingensis* as a species new to science.

## Introduction

The genus *Rhododendron* L. was established by Linnaeus in 1753, primarily based on stamen number and general morphological characters (Linnaeus 1753). In 1949, the German botanist Sleumer proposed a classification system comprising eight subgenera, based on leaf and floral anatomy, vegetative branching patterns, and secondary xylem structure (Sleumer 1949). Currently, it is recognized as one of the most species-rich woody genera, comprising eight subgenera, 12 sections, 71 subsections, and more than 1,300 species (Chamberlain et al. 1996; Xia et al. 2022). Recent molecular phylogenetic studies have further investigated the infrageneric relationships within the genus (Ma et al. 2022; Xia et al. 2022).

Geographically, the genus is widely distributed from the temperate regions of the Northern Hemisphere to tropical Southeast Asia, with the highest species richness occurring in East and Southeast Asia, and its center of diversity located in southern China (Geng 2014; Yan et al. 2015; Xia et al. 2022). China harbors an extremely rich diversity of *Rhododendron*, with the genus distributed across almost the entire country; more than 700 species are currently recognized (including varieties, subspecies, and forms), and new species continue to be described (Dai et al. 2020; Chang et al. 2021; Feng et al. 2023; Feng et al. 2024). Within this vast diversity, subgen. *Hymenanthes* is the second-largest subgenus of *Rhododendron* in terms of species number and includes a single section, sect. *Ponticum*, which is mainly distributed in Southeast Asia (Ma et al. 2022). Subsect. *Argyrophylla*, belonging to sect. *Ponticum*, comprises approximately 20 species and is endemic to China, occurring mainly in Yunnan, Guizhou, and Sichuan provinces (Fang et al. 2005; Ma et al. 2022).

In May 2019, during a field excursion in northwestern Guizhou, China, an unidentified *Rhododendron* taxon was discovered. Based on a combination of morphological characters, including 10 stamens, green and densely white-tomentose current-year branchlets, and its distinctive cliff habitat, the plant was preliminarily identified as a potential new species of subgen. *Hymenanthes*. The plants are small shrubs, with leaves crowded at branch apices, relatively small inflorescences bearing few flowers, short pedicels, and cylindric capsules. These traits, observed consistently in the field, prompted continuous field monitoring. Based on continuous field observations and specimen collections conducted between 2020–2025, we confirm that this *Rhododendron* from northwestern Guizhou represents a species new to science. Below, its distinctive morphological characters are described.

## Materials and methods

### Morphological study

Morphological characters of the new species, including vegetative and reproductive traits, were carefully observed and measured in the field. Subsequently, collected specimens were examined in the laboratory, and detailed measurements were taken using a stereomicroscope. Specimens were identified using relevant taxonomic literature (Fang et al. 2005; Geng 2014; Feng et al. 2024) and were compared with specimen images available in online herbaria, including the Kew Herbarium Catalogue (http://apps.kew.org/herbcat/navigator.do), PE (https://pe.ibcas.ac.cn/index.html), and JSTOR Global Plants (http://plants.jstor.org/). In addition, specimens from other online herbaria, such as CVH (https://www.cvh.ac.cn/index.php), RBGE (https://data.rbge.org.uk/search/herbarium/), and KUN (http://www.ui92.com/demo/html/1621/), were also examined. To ensure a robust taxonomic circumscription, the new species was compared with all 20 currently recognized species within subsect. *Argyrophylla* (Suppl. material 2: table S2). Furthermore, an identification key to the species of subsect. *Argyrophylla* was prepared by expanding the treatment in *Flora of China* (Fang et al. 2005) to accommodate the new species.

### Taxon sampling

A total of 15 samples representing 15 taxa were included in the phylogenetic analysis, comprising the newly discovered species *R. jiucaipingensis*, 12 species of subsect. *Argyrophylla*, and two outgroup species, *Cassiope selaginoides* (Ericaceae) and *R. simsii*. Detailed taxon information and accession numbers are provided in Suppl. material 1: table SS1. For the new species, *R. jiucaipingensis*, fresh leaf materials were collected in the field and used for DNA extraction. Whole-genome resequencing (WGS) data for the new species were newly generated for this study. These new data, together with the clean sequence reads of the remaining 14 taxa retrieved from the National Genomics Data Center (NGDC; accession number CRA005762), were aligned to the *R. prattii* reference genome (accession number GWHBHLU00000000) for variant calling (Ma et al. 2022). The resulting SNP dataset was subsequently utilized for all downstream phylogenetic and molecular analyses. Although all known species of subsect. *Argyrophylla* were surveyed, genomic sequences for the remaining eight species were unavailable and thus excluded from the molecular analysis. Nevertheless, these eight species exhibit distinct morphological differences from the new species, as detailed in the comprehensive morphological comparison (Suppl. material 2: table S2) and the identification key provided below.

### DNA extraction and sequencing

Total genomic DNA was extracted from fresh leaf material using the E.Z.N.A. Plant DNA Kit (Omega Bio-tek, Norcross, GA, USA) following the manufacturer’s instructions. DNA quality and concentration were assessed using 1% agarose gel electrophoresis and a NanoDrop spectrophotometer and a Quantus Fluorometer. For the new species, *R. jiucaipingensis*, a genomic library with an average insert size of 350 bp was constructed using the TruSeq Nano DNA Library Prep Kit (Illumina, San Diego, CA, USA) and sequenced on the Illumina NovaSeq X Plus platform at Shanghai Majorbio Bio-pharm Technology Co., Ltd. (Shanghai, China) with 150 bp paired-end reads. Approximately 6.76 Gb of raw data were generated. Raw reads were processed using fastp v0.23.2 (Chen et al. 2018) to remove adapter sequences and filter low-quality reads (Phred quality < 20). Quality control analysis showed that the sequencing error rate remained consistently low ( < 0.03%) across all read positions, with a Q30 score of 97.48% (yielding 6.74 Gb of clean data), indicating high sequence accuracy for subsequent phylogenetic analysis.

### Phylogenetic analysis

To reconstruct the phylogenetic relationships, the resulting high-quality clean reads from 15 samples were mapped to the reference genome using BWA-MEM v0.7.17 (Li 2013). SAM files were processed with SAMtools v1.6 (Danecek et al. 2021), sorted and indexed using SAMtools, and duplicate reads were removed via GATK v4.3.0 MarkDuplicates (McKenna et al. 2010; Van der Auwera et al. 2013). Variant calling and joint genotyping were performed using the HaplotypeCaller and GenotypeGVCFs modules in GATK. To ensure high-quality single nucleotide polymorphisms, hard filtering was applied via VariantFiltration (QD < 2.0, FS > 60.0, MQ < 40.0, SOR > 3.0, MQRankSum < –12.5, and ReadPosRankSum < – 8.0). While further filtering retained only biallelic sites with sequencing depths between 1/3× and 3× the average, the final alignment for phylogenetic analysis was not restricted to these variable sites. Instead, it included both the filtered biallelic SNPs and all invariant (constant) sites with zero missing data. This resulted in a final dataset of 5,870,491 nucleotide sites, which was converted to FASTA format using vcf2phylip v2.8 (Ortiz 2019). Because this comprehensive alignment retained 37.65% constant sites, the substitution model did not require additional ascertainment bias correction. A Maximum Likelihood (ML) tree was inferred using IQ-TREE v2.3.6 (Minh et al. 2020) under the TVM+F model, which was selected as the best-fit model via -m MFP. Because the alignment retained 37.65% constant sites, the substitution model did not require additional ascertainment bias correction. Node support was assessed via 1,000 Shimodaira–Hasegawa approximate likelihood ratio test (SH-aLRT) replicates (Guindon et al. 2010) and 5,000 ultrafast bootstrap (UFBoot) replicates (Hoang et al. 2018). Branches were considered strongly supported when SH-aLRT ≥ 80% and UFBoot ≥ 95%. The resulting consensus tree with node support values was visualized using the online tool ChiPlot (Xie et al. 2023).

## Results

### Rhododendron jiucaipingensis

Taxon classificationPlantaeEricalesEricaceae

Jian Xu & M. T. An sp. nov.

60744658-1932-5A25-822A-4AC56C6BC87B

urn:lsid:ipni.org:names:77380114-1

Figs 1, 2

#### Type.

**China** • Guizhou: Bijie City, Hezhang County, Xingfa Township, on cliffs, alt. 2,600 m, 27°00'08.86"N, 104°43'47.94"E, 8 May 2021, Jian Xu leg; holotype: GZAC! 20210508JCP001 • same data as for holotype; isotype: GZAC! 20210508JCP002

**Figure 1.**
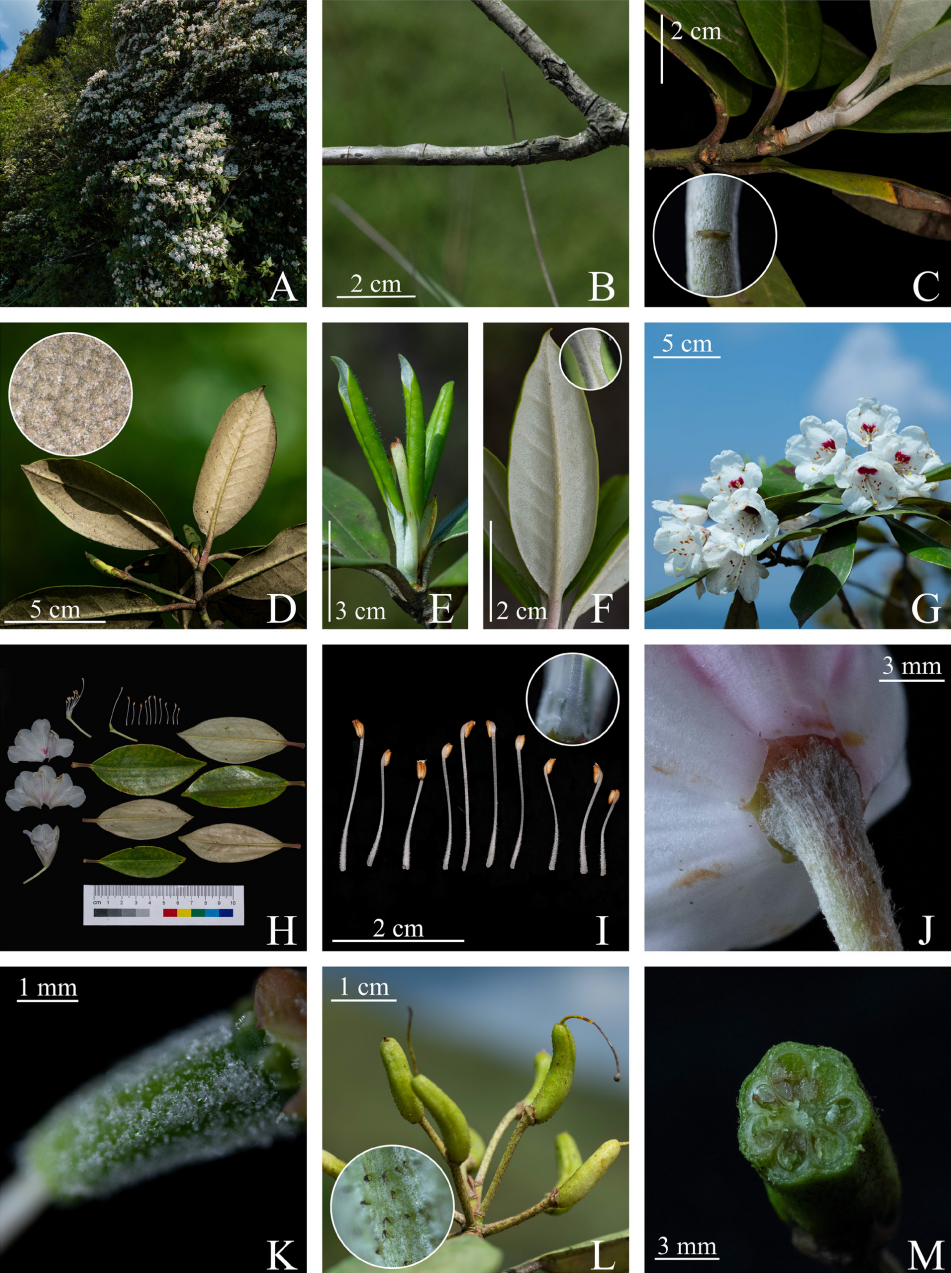
*Rhododendron jiucaipingensis* Jian Xu & M. T. An, sp. nov. **A**. Cliff habitat; **B**. Older branches; **C**. Current-year branchlet and its dense white tomentum (inset); **D**. Mature leaf and its short, brown tomentum on the abaxial surface (inset); **E**. Adaxial surface of young leaf; **F**. Abaxial surface of young leaf and long white glandular hairs of it (inset); **G**. Flowering plant; **H**. Anatomical diagram of the flower and leaf; **I**. Stamens and their white stiff hairs at the filament base (inset); **J**. Calyx; **K**. Ovary; **L**. Capsule and its colorless stellate hairs (inset); **M**. Capsule in longitudinal section (Photographs by Jian Xu)

#### Diagnosis.

*Rhododendron jiucaipingensis* belongs to subgen. *Hymenanthes* and is similar in morphology to *R. hypoglaucum* and *R. argyrophyllum*, but can be distinguished from *R. hypoglaucum* by its current-year branchlets and young petioles being densely covered with indumentum (**vs**. glabrous), and its pedicels being densely white-villous (**vs**. glabrous). It further differs from *R. hypoglaucum* in having fewer lateral veins (8–11 pairs **vs**. 10–14 pairs), an ovary sparsely covered with colorless stellate hairs (**vs**. glabrous), and typically 7-loculed capsules (**vs**. 6-loculed). Compared to *R. argyrophyllum*, the new species is distinguished by its acuminate leaf apex (**vs**. obtuse), fewer stamens (10 **vs**. 12–15), and the presence of colorless stellate hairs on the ovary (**vs**. white short tomentum). Additionally, *R. jiucaipingensis* is characterized by its restricted cliff habitat at higher elevations (2,400–2,700 m).

#### Description.

Evergreen shrubs, 1.5–3.0 m tall. Branches stout; current-year branchlets green, densely covered with white tomentum; older branches grayish brown, glabrescent. Leaves crowded at branch apices, thickly leathery, oblong-elliptic to oblanceolate-elliptic, 5.5–9.5 cm long, 2.5–4.0 cm wide; apex gradually acuminate, with a short apiculate tip; base gradually narrowed, cuneate; margins revolute; young leaves adaxially sparsely white-tomentose along the midrib, abaxially densely covered with a double-layered white indumentum, the upper layer consisting of long glandular hairs; mature leaves glabrescent adaxially; the superficial indumentum on the abaxial surface shed, leaving only a lower layer of short, brown tomentum; lateral veins 8–11 pairs, faintly visible adaxially and conspicuously raised abaxially; petioles cylindric, 1.0–1.5 cm long, grooved adaxially, densely covered with long white glandular hairs when young, becoming glabrous at maturity. Inflorescences terminal, umbellate-racemose, with 4–9 flowers; peduncle 1.0–1.3 cm long, glabrous; pedicels 1.5–2.5 cm long, densely covered with long white villous hairs; calyx triangular, ca. 1.5 mm long, 5-lobed or with lobes partially connate; corolla funnelform-campanulate, 2.0–3.0 cm long, 2.0–3.0 cm in diameter in the upper part, white, the tube with pink fasciculate blotches and spots, 5-lobed, lobes orbicular, ca. 1 cm long, ca. 1.5 cm wide, apex rounded and emarginate; stamens 10, 1.5–2.0 cm long, unequal; filaments linear, densely covered with white stiff hairs at the base, anthers oblong-elliptic, 0.2–0.3 cm long; pistil 2.5–3.0 cm long, glabrous; ovary cylindric, sparsely covered with colorless stellate hairs; style ca. 2.5 cm long, glabrous; stigma slightly dilated. Capsules cylindric, curved, 1.2–2.0 cm long, ca. 0.6 cm in diameter, sparsely covered with colorless stellate hairs, usually 7-loculed, occasionally 6- or 8-loculed.

#### Phenology.

Flowering in May, fruiting in June.

#### Etymology.

The specific epithet *jiucaipingensis* refers to the type locality, Xingfa Township (Jiucaiping), Hezhang County, Bijie City, Guizhou, China.

#### Vernacular name.

The Chinese name is jiǔ cài píng dù juān (韭菜坪杜鹃).

#### Distribution and habitat.

**Figure 2.**
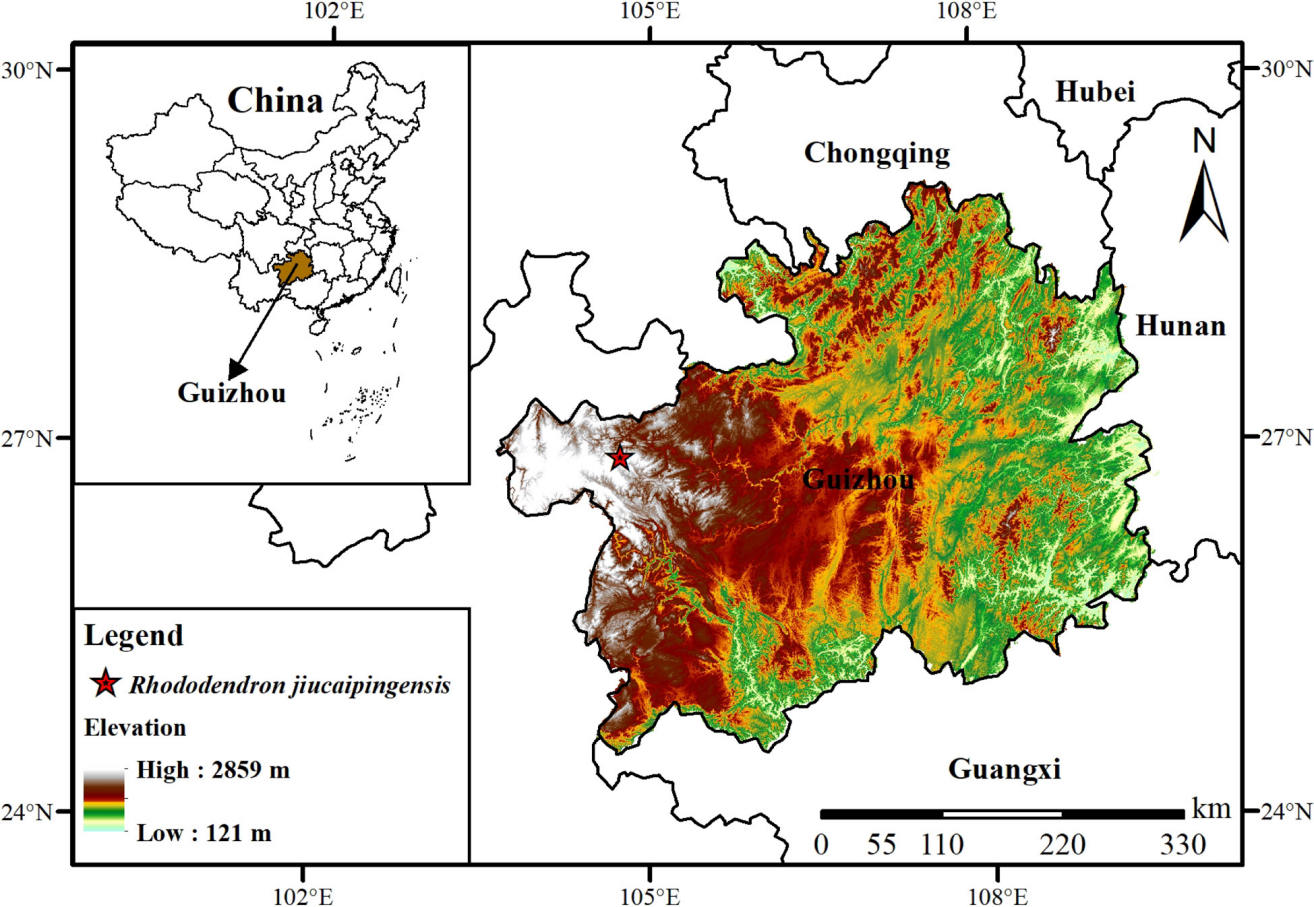
Known geographic distribution of *Rhododendron jiucaipingensis* Jian Xu & M. T. An, sp. nov.

This species is currently known only from western Guizhou, China (Fig. 2), where it grows on cliffs at elevations of 2,400–2,700 m. The type specimens were collected from Xingfa Township (Jiucaiping), Hezhang County, Bijie City, Guizhou, China.

#### Conservation status.

*Rhododendron jiucaipingensis* is known only from 21 extremely restricted populations in Jiucaiping, Xingfa Township, totaling 103 mature individuals. The species has an extremely restricted Area of Occupancy (AOO < 10 km^2^, estimated using a 2 × 2 km grid) and occurs at a single location according to IUCN criteria (IUCN Standards and Petitions Committee 2024). Populations may be vulnerable to habitat degradation due to their cliffside habitat and potential anthropogenic activities, including climate change and collection. With such a small number of mature individuals, the species qualifies as Endangered [EN] under Criterion D1. Although a continuing decline has not been directly observed, the extremely limited AOO, single-location occurrence, projected threats to habitat quality, and small population size meet the IUCN Red List criteria for Endangered [EN] under Criteria B2ab(iii) + D1.

#### Additional specimens examined.

**China** • Guizhou: Bijie City, Hezhang County, Xingfa Township, on cliffs, alt. 2,600 m, 27°00'02.48"N, 104°43'54.92"E, 13 May 2021, Jian Xu leg.; paratype: GZBG! XJ20210513001• same data as for preceding; paratype: GZBG! XJ20210513002.

#### Similar species.

*Rhododendron jiucaipingensis* is morphologically and phylogenetically most similar to *R. hypoglaucum* and *R. argyrophyllum*, which are also distributed in the karst regions of Southwest China. All three species are endemic to the karst regions of Southwest China and share white to pinkish campanulate corollas. However, *R. jiucaipingensis* is readily distinguished by its densely white-tomentose young branchlets, young petioles with long white glandular hairs, and mature leaf abaxial surfaces with short, brown tomentum. Additionally, the white stiff hairs at the filament base and colorless stellate hairs on the ovary and capsule serve as key diagnostic features. Detailed morphological comparisons are summarized in Table 1.

**Table 1.** Morphological comparison of *R. jiucaipingensis*, *R. hypoglaucum* and *R. argyrophyllum*.

**Characters**	***R. jiucaipingensis***	***R. hypoglaucum***	***R. argyrophyllum***
Altitude (m)	2,400–2,700	1,500–2,100	1,600–2,300
Plant height (m)	1.5–3.0	3.0–10.0	3.0–7.0
Young branch color	Green	Light green	Light green or purplish green
Indumentum of young branches	densely covered with white tomentum	glabrous	often glabrous
Leaf size (cm)	5.5–9.5 × 2.5–4.0	6.0–10.0 × 2.0–3.5	8.0–13.0 × 2.0–4.0
Leaf apex	acuminate	acute	obtuse
Indumentum of abaxial leaf surface at maturity	brown short tomentum	silvery appressed hairs	silvery appressed hairs
Lateral veins (pairs)	8–11	10–14	ca. 12–14
Petiole indumentum	densely covered with long glandular hairs when young, glabrous at maturity	glabrous	pubescent when young, becoming glabrous
Pedicel indumentum	densely covered with white villous hairs	glabrous	sparsely white floccose hairs
Calyx length (mm)	ca. 1.5	ca. 2.0	—
Corolla color	white	creamy white to pink	cream white or pink
Corolla markings	pink	deep red to purplish red	purple
Stamens (no.)	10	10	12–15
Stamen length (cm)	1.5–2.0	1.5–3.0	1.2–2.5
Indumentum at filament base	densely covered with white stiff hairs	white tomentum	finely puberulent
Ovary indumentum	sparsely covered with colorless stellate hairs	glabrous or sparsely hairy at apex	white short tomentum
Capsule length (cm)	1.2–2.0	2.0–2.5	1.8–2.5
Capsule indumentum	sparsely covered with colorless stellate hairs	glabrous	white short tomentum or glabrous
Locules (no.)	(6–)7(–8)	6	—

#### Molecular phylogenetic evidence.

**Figure 3.**
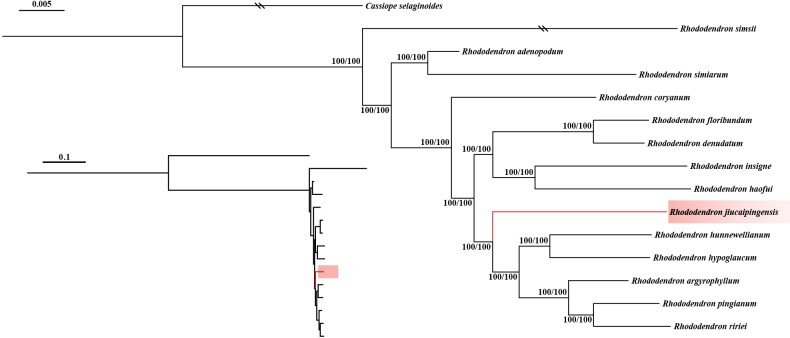
The ML tree of *Rhododendron* subsect. *Argyrophylla* inferred from SNP data. Numbers at nodes represent SH-aLRT and UFBoot support values. Double slashes denote truncated branches. The new species, *R. jiucaipingensis*, is indicated in red.

The ML analysis, based on a comprehensive whole-genome SNP dataset, yielded a highly resolved phylogenetic framework (Fig. 3). Within this topology, *R. jiucaipingensis* is robustly recovered as a distinct and independent evolutionary lineage within subsect. *Argyrophylla*. The new species is strongly supported (SH-aLRT = 100%, UFBoot = 100%) as sister to a monophyletic clade comprising five species: *R. hunnewellianum*, *R. hypoglaucum*, *R. argyrophyllum*, *R. pingianum*, and *R. ririei*. Despite its close phylogenetic affinity to this five-species group, *R. jiucaipingensis* exhibits significant genetic divergence, occupying a well-supported terminal branch that underscores its long-term evolutionary isolation. This unambiguous molecular evidence, coupled with the clear morphological discontinuities observed (Table 1), provides compelling support for the recognition of *R. jiucaipingensis* as a distinct new species within subsect. *Argyrophylla*.

##### Key to the species of the *Rhododendron* sect. *Argyrophylla*, expanded from Flora of China (2005)

**Table:** 

1	Leaves adaxially bullate-rugose, abaxially lanate; pedicels 1–1.5 cm	**2**
–	Leaves adaxially flat, abaxially appressed-indumentum; pedicels 2–4 cm	**4**
2	Leaves 7–8 cm, abaxially thick pale gray; corolla white	***R. farinosum***
–	Leaves 10–15 cm, abaxially loose tomentose; corolla dark red	**3**
3	Leaf base rounded, asymmetrical; corolla rose	***R. denudatum***
–	Leaf base cuneate; veins raised; corolla red to purplish red	***R. floribundum***
4	Leaves abaxially silvery-white or grayish-white	**5**
–	Leaves abaxially pale brown or muddy-gray	**12**
5	Leaves 6–13 cm long; pedicels hairy and glandular	**6**
–	Leaves 7–15 cm long; pedicels glabrous or varied	**8**
6	Indumentum double-layered, upper layer loose; leaves narrowly oblanceolate	***R. hunnewellianum***
–	Indumentum single-layered; pedicels densely long-stipitate glandular	**7**
7	Leaves obovate; ovary glandular; style glabrous	***R. adenopodum***
–	Leaves lanceolate; ovary glandular and stiff-hairy; style base glandular	***R. ebianense***
8	Corolla 4–6 cm, purplish red, with nectar pouches	***R. ririei***
–	Corolla 2–3.5 cm, pink to white, without nectar pouches	**9**
9	Indumentum double-layered, upper layer furfuraceous; filaments glabrous	***R. pingianum***
–	Indumentum single-layered; filaments hairy at base	**10**
10	Ovary white tomentose	***R. argyrophyllum***
–	Ovary glabrous	**11**
11	Abaxial indumentum dull; pedicels slightly hairy	***R. coryanum***
–	Abaxial indumentum shiny; pedicels glabrous	***R. hypoglaucum***
12	Leaves 1.5–2 cm wide, oblanceolate; rachis 2–4 mm	***R. oblancifolium***
–	Leaves 2–5 cm wide; rachis 1–3.5 cm	**13**
13	Corolla dark red, campanulate, with nectar pouches; petioles ca. 1 cm	***R. brevipetiolatum***
–	Corolla white or pink, without nectar pouches; petioles 1.5–2.5 cm	**14**
14	Leaf apex subacute to obtuse; pedicels hairy	**15**
–	Leaf apex acuminate; pedicels and rachis varied (hairy/glandular)	**18**
15	Leaves leathery, apex subacute to acute	**16**
–	Leaves thickly leathery, apex somewhat obtuse	**17**
16	Shoots and petioles hairy; stamens 10–12; ovary brown tomentose	***R. formosanum***
–	Shoots and petioles glabrous; stamens 18–20; ovary white lanate	***R. haofui***
17	Leaves 8–15 cm, adaxially often glaucous; style base yellow tomentose	***R. fangchengense***
–	Leaves 5.5–10 cm, adaxially not glaucous; ovary and style base glandular	***R. simiarum***
18	Shoots with persistent bud scales; rachis, pedicels and ovary glandular	***R. thayerianum***
–	Shoots without bud scales; ovary hairy; style glabrous	**19**
19	Rachis glabrous; pedicels hairy; filaments hairy	**20**
–	Rachis hairy; filaments glabrous; pedicels glabrous or glandular	***R. longipes***
20	Leaves 8–13 cm, abaxially silvery-white; stamens 13–15	***R. insigne***
–	Leaves 5.5–9.5 cm, abaxially with persistent brown tomentum; stamens 10	***R. jiucaipingensis***

## Supplementary Material

XML Treatment for Rhododendron jiucaipingensis

## Additional information

### Artificial Intelligence (AI) use

The authors accept full responsibility for the content of the manuscript, including the disclosure of any use of AI.

No AI tools were used in the preparation of this manuscript.

### Author ORCIDs

H-F. Hu https://orcid.org/0009-0008-4761-8242

J. Xu https://orcid.org/0000-0002-0714-0917

M-T. An https://orcid.org/0000-0003-3886-0287

J-H. Yu https://orcid.org/0000-0003-1765-8557

### Data availability

The data that support the findings of this study are available in the main text or Supplementary Information. Whole-genome sequencing data (clean reads) have been deposited in the Genome Sequence Archive (GSA) at the National Genomics Data Center (NGDC), China National Center for Bioinformation (CNCB), under accession number CRA042072 (https://ngdc.cncb.ac.cn/gsa).

## Supplementary materials

Supplementary material 1 Supplementary table SS1 Data type docx Explanation note The GenBank accession numbers for DNA sequences used in this study. https://binary.pensoft.net/file/1650602This dataset is made available under the Open Database License (http://opendatacommons.org/licenses/odbl/1.0/). The Open Database License (ODbL) is a license agreement intended to allow users to freely share, modify, and use this Dataset while maintaining this same freedom for others, provided that the original source and author(s) are credited. Hong-Fen Hu

Supplementary material 2 Supplementary table S2 Data type xlsx https://binary.pensoft.net/file/1650603This dataset is made available under the Open Database License (http://opendatacommons.org/licenses/odbl/1.0/). The Open Database License (ODbL) is a license agreement intended to allow users to freely share, modify, and use this Dataset while maintaining this same freedom for others, provided that the original source and author(s) are credited. Hong-Fen Hu, Jian Xu, Ming-Tai An, Jiang-Hong Yu, Xing Lu
