# Supplementary material for: Rhododendron jiucaipingensis (Ericaceae), a new species from Guizhou, China
Source: PhytoKeys. 2026 May 19;275:15–26. doi: 10.3897/phytokeys.275.183519 (PMC13213387; doi:10.3897/phytokeys.275.183519)
Supplement: Supplementary material 1 — Supplementary table SS1 [file phytokeys-275-015_article-183519__-s001.docx]

**Appendix 1**

**Table S1.** The GenBank accession numbers for DNA sequences used in this study.

| **Species** | **GenBank Accession number** |
| --- | --- |
| *Empetrum nigrum* | GU176670 |
| *Rhododendron hunnewellianum* | SRR19401175 |
| *Rhododendron denudatum* | MW899369 |
| *Rhododendron adenopodum* | EU087299 |
| *Rhododendron insigne* | MT779575 |
| *Rhododendron argyrophyllum* | EU087302 |
| *Rhododendron coryanum* | KM606240 |
| *Rhododendron thayerianum* | MT779577 |
